# An international working group consensus report for the prioritization of molecular biomarkers for Ewing sarcoma

**DOI:** 10.1038/s41698-022-00307-2

**Published:** 2022-09-17

**Authors:** David S. Shulman, Sarah B. Whittle, Didier Surdez, Kelly M. Bailey, Enrique de Álava, Jason T. Yustein, Adam Shlien, Masanori Hayashi, Alexander J. R. Bishop, Brian D. Crompton, Steven G. DuBois, Neerav Shukla, Patrick J. Leavey, Stephen L. Lessnick, Heinrich Kovar, Olivier Delattre, Thomas G. P. Grünewald, Cristina R. Antonescu, Ryan D. Roberts, Jeffrey A. Toretsky, Franck Tirode, Richard Gorlick, Katherine A. Janeway, Damon Reed, Elizabeth R. Lawlor, Patrick J. Grohar

**Affiliations:** 1 Dana-Farber/Boston Children’s Cancer and Blood Disorders Center, Harvard Medical School, Boston, MA USA; 2 Texas Children’s Cancer and Hematology Centers, Department of Pediatrics, Baylor College of Medicine, Houston, TX USA; 3 Bone Sarcoma Research Laboratory, Balgrist University Hospital, University of Zurich, Zurich, Switzerland; 4 Department of Pediatrics, University of Pittsburgh School of Medicine, Pittsburgh, PA USA; 5 Institute of Biomedicine of Sevilla (IBiS), Virgen del Rocio University Hospital/CSIC/University of Sevilla/CIBERONC/Department of Normal and Pathological Cytology and Histology, School of Medicine, University of Seville, Seville, Spain; 6 Texas Children’s Cancer and Hematology Center and The Faris D. Virani Ewing Sarcoma Center, Baylor College of Medicine, Houston, TX USA; 7 Department of Laboratory Medicine and Pathobiology/Department of Paediatric Laboratory Medicine/Program in Genetics and Genome Biology, The Hospital for Sick Children, Toronto, ON Canada; 8 Department of Pediatrics, University of Colorado Anschutz Medical Campus and Center for Cancer and Blood Disorders, Children’s Hospital Colorado, Aurora, CO USA; 9 Greehey Children’s Cancer Research Institute and Department of Cell Systems and Anatomy, University of Texas Health at San Antonio, San Antonio, TX USA; 10 Department of Pediatrics, Memorial Sloan Kettering Cancer Center, New York, NY USA; 11 Department of Pediatrics, UT Southwestern Medical Center, Dallas, TX USA; 12 Center for Childhood Cancer and Blood Diseases, Abigail Wexner Research Institute at Nationwide Children’s Hospital, and the Division of Pediatric Heme/Onc/BMT, The Ohio State University College of Medicine, Columbus, OH USA; 13 St. Anna Children´s Cancer Research Institute (CCRI) and Department Pediatrics Medical University of Vienna, Vienna, Austria; 14 INSERM U830, Diversity and Plasticity of Childhood Tumors Lab, PSL Research University, SIREDO Oncology Center, Institut Curie Research Center, Paris, France; 15 Hopp-Children’s Cancer Center (KiTZ), Heidelberg/Division of Translational Pediatric Sarcoma Research, German Cancer Research Center (DKFZ), German Cancer Consortium (DKTK)/Institut of Pathology, Heidelberg University Hospital, Heidelberg, Germany; 16 Department of Pathology, Memorial Sloan Kettering Cancer Center, New York, NY USA; 17 Center for Childhood Cancer and Blood Disease, Nationwide Children’s Hospital and The Ohio State University, Columbus, OH USA; 18 Departments of Oncology and Pediatrics, Georgetown University, Washington, DC USA; 19 Univ Lyon, Universite Claude Bernard Lyon 1, INSERM 1052, CNRS 5286, Cancer Research Center of Lyon, Centre Leon Berard, F-69008 Lyon, France; 20 Division of Pediatrics, MD Anderson Cancer Center, Houston, TX USA; 21 Department of Individualized Cancer Management, Moffitt Cancer Center, Tampa, FL USA; 22 Seattle Children’s Research Institute, University of Washington Medical School, Seattle, WA USA; 23 Center for Childhood Cancer Research, Children’s Hospital of Philadelphia, University of Pennsylvania, Perelman School of Medicine, Philadelphia, PA USA

**Keywords:** Sarcoma, Cancer genetics, Prognostic markers, Molecular medicine

## Abstract

The advent of dose intensified interval compressed therapy has improved event-free survival for patients with localized Ewing sarcoma (EwS) to 78% at 5 years. However, nearly a quarter of patients with localized tumors and 60–80% of patients with metastatic tumors suffer relapse and die of disease. In addition, those who survive are often left with debilitating late effects. Clinical features aside from stage have proven inadequate to meaningfully classify patients for risk-stratified therapy. Therefore, there is a critical need to develop approaches to risk stratify patients with EwS based on molecular features. Over the past decade, new technology has enabled the study of multiple molecular biomarkers in EwS. Preliminary evidence requiring validation supports copy number changes, and loss of function mutations in tumor suppressor genes as biomarkers of outcome in EwS. Initial studies of circulating tumor DNA demonstrated that diagnostic ctDNA burden and ctDNA clearance during induction are also associated with outcome. In addition, fusion partner should be a pre-requisite for enrollment on EwS clinical trials, and the fusion type and structure require further study to determine prognostic impact. These emerging biomarkers represent a new horizon in our understanding of disease risk and will enable future efforts to develop risk-adapted treatment.

## Introduction

Ewing sarcoma (EwS) is a rare, aggressive sarcoma with a peak incidence in adolescents and young adults (AYAs). Primary tumors may arise in the soft tissue or bone, and staging differentiates patients with localized vs. metastatic disease. All patients receive multi-agent intensive chemotherapy and local control^1–4^. Despite this treatment, over 20% of patients with localized disease and 60–80% of patients with metastatic disease relapse with lethal disease, while long-term survivors are left with a significant burden of late effects^1,5–9^. Beyond the presence of metastatic disease, clinical features may not be fully sufficient to develop risk-stratified therapy.

Improved outcomes have come at a cost, with rates of second malignant neoplasms reported between 10 and 20.5% at 30 years^10,11^. Further, other late effects, including anthracycline-induced cardiotoxicity, negatively impact quality of life and long-term survival^11^. Therefore, future efforts to improve outcomes for patients with EwS must aim to identify those patients with a poor prognosis who would benefit from additional or alternative therapies and those patients who may be candidates for a reduction in therapy. Many of the previously characterized prognostic clinical features have proven inadequate to identify sufficiently high- or low-risk subgroups to warrant testing intensified or de-intensified therapies, especially when evaluated in the context of contemporary intensified therapy^6,12,13^. Therefore, there is a clear need for molecular biomarkers to better delineate disease subgroups either alone or in combination with clinical features.

**Fig. 1:**
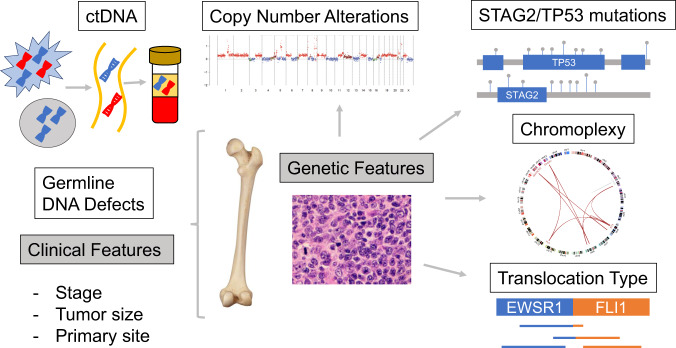
Schematic representation of potential prognostic biomarkers. In this review we evaluate multiple molecular biomarkers with the potential to inform testing of risk-stratified therapy on a future therapeutic trial. Here we provide a graphical representation of the biomarkers evaluated.

To evaluate potential molecular prognostic biomarkers, we organized an international working group of disease experts to evaluate the most promising and clinically mature candidates in EwS and update a prior consensus published in 2013^14^. Our working group reviewed the prognostic significance of a select group of contemporary molecular biomarkers with strong pre-clinical evidence including translocation subtype and mechanism of formation; *STAG2* loss and *TP53* pathogenic mutations; copy number variants; tumor mutational burden (TMB); circulating tumor DNA (ctDNA); and germline DNA defects (Fig. 1). We evaluated the current level of evidence for each available biomarker and identified molecular features that this expert panel agrees should be assessed in all future phase II/III studies. Biomarker data were evaluated in context of the relevant treatment regimen, with the understanding that 5-drug interval compressed therapy is now considered the preferred chemotherapy backbone in Europe and North America (Supplemental Table 1)^1,4^. These molecular biomarkers include those important for proper diagnosis and several that warrant prospective evaluation to validate prognostic value. This review is not intended to be comprehensive of all biomarkers in EwS, but rather focus on those judged to be most promising to inform risk-adapted therapy in the near future.

## Current definition of EwS

### Molecular diagnostics

The latest 2020 WHO Classification of Tumors of Soft Tissue and Bone includes four categories for round cell sarcomas: EwS; round cell sarcoma with *EWSR1*::non-*ETS* fusions; *CIC-*rearranged sarcomas; and sarcomas with *BCOR* gene alterations^15^. Based on a recent international survey of pathologists and oncologists, most respondents defined EwS as harboring *FET::ETS* gene family fusions and agreed this group should define a primary therapy arm for a future frontline clinical trial^16^. However, a consensus was not met on how to classify the remaining round cell sarcomas with molecular variants. This survey highlights the need for centralized molecular test standardization within clinical trials to provide a uniform approach in diagnosis, classification, and treatment of these patients.

Classic EwS is characterized by solid sheets of monomorphic round cells with ill-defined cell borders, scant, clear cytoplasm and round, uniform nuclei with open or fine chromatin. It typically lacks nuclear pleomorphism, spindling or epithelioid morphology. Immunohistochemically, most EwS tumors show diffuse and strong membranous CD99 positivity as well as NKX2.2 and FLI1 staining^17^. The defining molecular feature of the tumor is the characteristic *FET::ETS* fusion involving: *EWSR1::FLI1* (70–80%) and *EWSR1/FUS::ERG* fusions (15%), followed by *EWSR1/FUS::FEV* (5%), and *EWSR1::ETV1/4* (1%)^18^. Ewing sarcoma (EwS) with *ERG* fusions may display a more variable spectrum of histologies^18^, and due to the unbalanced translocation, FISH testing for *EWSR1* gene rearrangements may show false negative results in more than half of the cases^19^. Such patients may require RNA or DNA sequencing approaches for fusion confirmation.

The incidence of sarcoma with *EWSR1*::non-ETS fusions accounted for 6% of the 240 patients in a recently analyzed large cohort of patients with round cell sarcomas with *EWSR1* or *FUS* fusions^18^. Alternative *EWSR1* rearrangements present diagnostic challenges with therapeutic implications and highlight the underperformance of *EWSR1* break-apart FISH as a sole means for molecular diagnosis. For example, *EWSR1::NFATc2* and *EWSR1::PATZ1* rearranged tumors are epigenetically and genomically distinct from EwS with canonical fusions^20,21^. Although most tumors show positivity for CD99, a small subset of cases show focal or negative staining^18^. In addition, *EWSR1::PATZ1* sarcomas exhibit a divergent morphology with round and spindle cell features and a polyphenotypic immunoprofile which may pose significant diagnostic pitfalls and simulate other sarcoma types^22,23^. *EWSR1::NFATc2* positive tumors are more common in older patients, characterized by monomorphic round to epithelioid cells in anastomosing cords and abundant myxohyaline to collagenous extracellular matrix. They harbor *EWSR1* rearrangements and gains/amplifications which may serve as a diagnostic hint^24–26^. The overall prognosis of these patients is poor^18,27^.

*CIC::DUX4* gene fusion, resulting from either t(4;19) or t(10;19) translocation, is the most common genetic abnormality detected in two-thirds of *FET*-negative round cell sarcomas^28^. *CIC*-rearranged sarcomas occur most commonly in young adults and have an inferior outcome with a 5-year survival of only 43% vs. 77% for EwS (*P* = 0.002) in one series of 115 patients^29^. Tumors have a predilection for soft tissue and show a variable round cell phenotype, admixed with epithelioid, spindle and myxoid stroma components with immunopositivity for WT1 and ETV4 and variable CD99 expression^29^. Molecular studies have highlighted the underperformance of FISH and RNAseq methods in diagnosing sarcomas with *CIC* gene abnormalities^30^. Similarly, despite histological similarities, the BCOR family of tumors demonstrate distinctive clinical presentations and outcomes, including an overall favorable prognosis for patients with *BCOR::CCNB3-*positive tumors^31^, and a highly aggressive behavior for *BCOR* ITD round cell sarcomas^32^.

In summary, the presence of variant translocations may be difficult to detect or lead to the misclassification of tumors as EwS. Further, many variant translocations are not readily detectable by morphology or standard FISH. DNA and RNA-based sequencing approaches may be required in some cases for translocation identification/confirmation. Therefore, National Comprehensive Cancer Network (NCCN) guidelines recommend assessment of sarcoma pathology in a center with access to specialized testing, as these diagnoses are nuanced. Nevertheless, because the biology, outcomes and responsiveness to EwS-based treatment are distinct among patients with variant translocations, clinical trials in EwS should assess the primary objective in patients with *FET::ETS* fusion-positive EwS. Future phase II/III clinical studies must include molecular diagnostics for fusion partners to accurately classify patients as having EwS to allow the interpretation of trials designed for this tumor. Testing should aim to identify both fusion partners and preferably be performed at a centralized laboratory.

## Clinical prognostic features in EwS

For patients with newly diagnosed EwS, stage remains the strongest prognostic factor. Patients presenting with metastatic disease have poor outcomes with a 2-year event-free survival (EFS) of 20–40%^5,33^. Among patients with metastatic disease, multiple studies have demonstrated that patients with isolated pulmonary metastatic disease have more favorable outcomes compared to patients with extrapulmonary metastasis^34–36^. In contrast, for patients treated on the most recent Children’s Oncology Group (COG) frontline trial for localized disease, 5-year EFS was 78%^6^.

Among the 70% of patients who present with localized tumors, the evidence for clinically useful prognostic factors is variable. In Europe, tumor size at presentation and histologic response have been used in clinical trials to differentiate patients with localized tumors into standard risk and high-risk groups^37^. Tumor size at presentation was prognostic in multiple studies^38–40^. Tumor volume of ≥200 mL was used on the recent European upfront trials to identify patients with high-risk localized disease. While prognostic on the most recent COG trial for patients with non-metastatic disease AEWS1031, patients with tumors ≥8 cm or ≥200 mL still had a 5-year EFS of 70% or greater and thus, in the context of the now international standard interval compressed 5-drug chemotherapy, tumor size holds modest prognostic impact^6^.

Response to therapy as assessed by histologic response following induction therapy is used to identify patients with high-risk localized disease in European trials^41,42^. However, in the context of 5-drug interval compressed chemotherapy, tumor necrosis was modestly prognostic with data demonstrating a 5-year EFS of 81% (95% CI 3–87%) and 75% (95% CI 4–81%) for patients with no viable tumor vs. any viable tumor at local control (*P* = 0.055)^6^. The evidence for radiologic response is variable^43^.

In addition, age at diagnosis, pelvic primary site, race, ethnicity, and sex have all been shown to carry modest prognostic significance^12,13,34,39,44^. These historic studies are informative but cannot be fully generalized because these patients were treated with historic chemotherapy regimens, and eligibility relied upon histologic diagnosis and did not necessarily exclude patients with variant EwS translocations. Therefore, in the context of the current international standard of interval compressed therapy, these clinical risk factors provide only modest prognostic information and have not been used to identify patients with notably high or low-risk disease for testing risk-adapted therapy. Nevertheless, clinical features may be useful to identify low- and high-risk subgroups in patients with *FET::ETS* fusions when considered in the context of contemporary therapy and the molecular biomarkers described below.

## Summary of evidence for molecular biomarkers for risk-stratification at diagnosis

### Evaluation strategy for available biomarkers

The molecular biomarkers described in this review were evaluated by an expert working group from the North America and Europe. The relevant literature for each molecular biomarker was reviewed by the group who determined the strength of the available literature, viable assays through which the biomarker can be assessed, and whether the current state of evidence suggests the biomarker should be: (1) assessed only in the research space; (2) requires further investigation in a larger cohort; (3) requires prospective validation with a pre-defined statistical plan; or (4) can be used to define eligibility or disease subgroups for therapeutic stratification on a prospective trial. Given this was an international working group, strict use of biomarker language specific to regional regulatory bodies was avoided.

### Translocation and chromoplexy

The FET-ETS fusion protein is the defining molecular feature of EwS (see molecular diagnosis section above). Most commonly, *EWSR1::FLI1* forms from the t(11;22)(q24;12) chromosomal translocation joining exon 7 of *EWSR1* to either exon 6 (type 1; 60% of cases) or exon 5 (type II; 25% of cases) of *FLI1*^45^. Alternative fusions occur between exons 7, 9, 10 of *EWSR1* or exons 4–8 of *FLI;* including a subset of tumors with a cryptic exon 8 (and intronic breakpoint) that is universally spliced out to yield a mature functional type I *EWSR1::FLI1*^46^. In addition, alternative *FET::ETS* fusions have been described either involving the *EWSR1* family member *FUS* or more commonly with other ETS family members such as *ERG*, *ETV1*, *ETV4*, and *FEV*^47–52^.

Early studies suggested that a type I *EWSR1::FLI1* fusion might be prognostic and suggested a higher probability of relapse free survival for 31 patients with type I fusions relative to 24 patients with alternative fusions (RFS = 0.72 ± 0.1 vs. 0.21 ± 0.12; *p* = 0.04)^53^. A follow-up multivariate analysis of 99 patients with EwS supported this idea and reported a relative risk (RR) of 0.37 (*P* = 0.014) for type I fusions relative to all other fusion types (RR = 0.32; *p* = 0.034)^54^. However, a larger data set of 119 patients treated with 5-drug chemotherapy in the COG failed to demonstrate a difference in either EFS or OS for type I vs. non-type I *EWSR1::FLI1* fusions^55^. Consistent with this observation, the largest analysis to date of 565 patients treated on the Euro-E.W.I.N.G. 99 trial also failed to demonstrate a difference in relapse or death among patients with type I, type II or *EWSR1::ERG* fusions (*P* = 0.95 and *P* = 0.83)^56^. However, there was a slight, albeit non-significant increase in the risk of relapse or progression (HR, 1.38; 95% CI, 0.96–2.0 *P* = 0.1) or death (HR, 1.48; 95% CI, 0.98–2.2; *P* = 0.07) in 91 patients with *EWSR1::FLI1* fusions that were called “EFx” and excluded type I, II and *EWSR1::ERG* fusions. This group was not captured in the COG analysis and includes the type III fusion protein (*EWSR1* exon 10 fused to *FLI1* exon 6). Importantly, the additional *EWSR1* exons lead to the inclusion of functional IQ and RGG domains that could influence sensitivity to either standard or targeted therapy^57,58^. Therefore, while a type I fusion is unlikely to be prognostic, the EFx subgroup (inclusive of type III fusions) remains an open question.

An intriguing possibility is that the mechanism of translocation could be prognostic. Although chromosomal translocations form by multiple mechanisms, in some tumors they result from catastrophic genomic events like chromosome shattering, called chromothripsis, or aberrant repair of loop-structures, known as chromoplexy^59–61^. In EwS, the occurrence of chromoplexy was found to be quite common, occurring in 42% of cases (52/124)^62^. This included all *EWSR1::ERG* rearrangements as well as a subset of canonical *EWSR1::FLI1* fusions. Importantly, the presence of chromoplexy increased the likelihood of relapse (54% vs. 30%, *p* < 0.05)^62^. Consistent with the higher rate of relapse, there was also an association with poor prognostic molecular features such as higher mutational burden and *TP53* mutation (see TP53 section), but not *STAG2* or *CDKN2A* mutations^62^. Further, 504 differentially expressed genes distinguished EwS tumors formed by chromoplexy from “simple EwS” (*P* < 0.001), suggesting the activation of distinct biological pathways.

In summary, while the type I fusion is unlikely to be prognostic in the setting of interval compressed therapy, the type EFx subtype remains an open question. Further, processes that create fusions through catastrophic chromosomal events like chromoplexy lead to altered transcriptional profiles and may identify patients with inferior outcomes. Finally, as described above, variant translocations in tumors with inferior outcomes, such as *EWSR1::NFATc2* and *CIC::DUX4* fusions, should be considered distinct biological entities and evaluated separately in clinical trials^29,63^. Therefore, these data support the need for centralized translocation testing for patients with EwS enrolled on clinical studies, ideally with an assay that can identify fusions generated by catastrophic chromosomal events such as chromoplexy.

### Copy number variants 1q gain, 16q loss, 8q gain, other

**Fig. 2:**
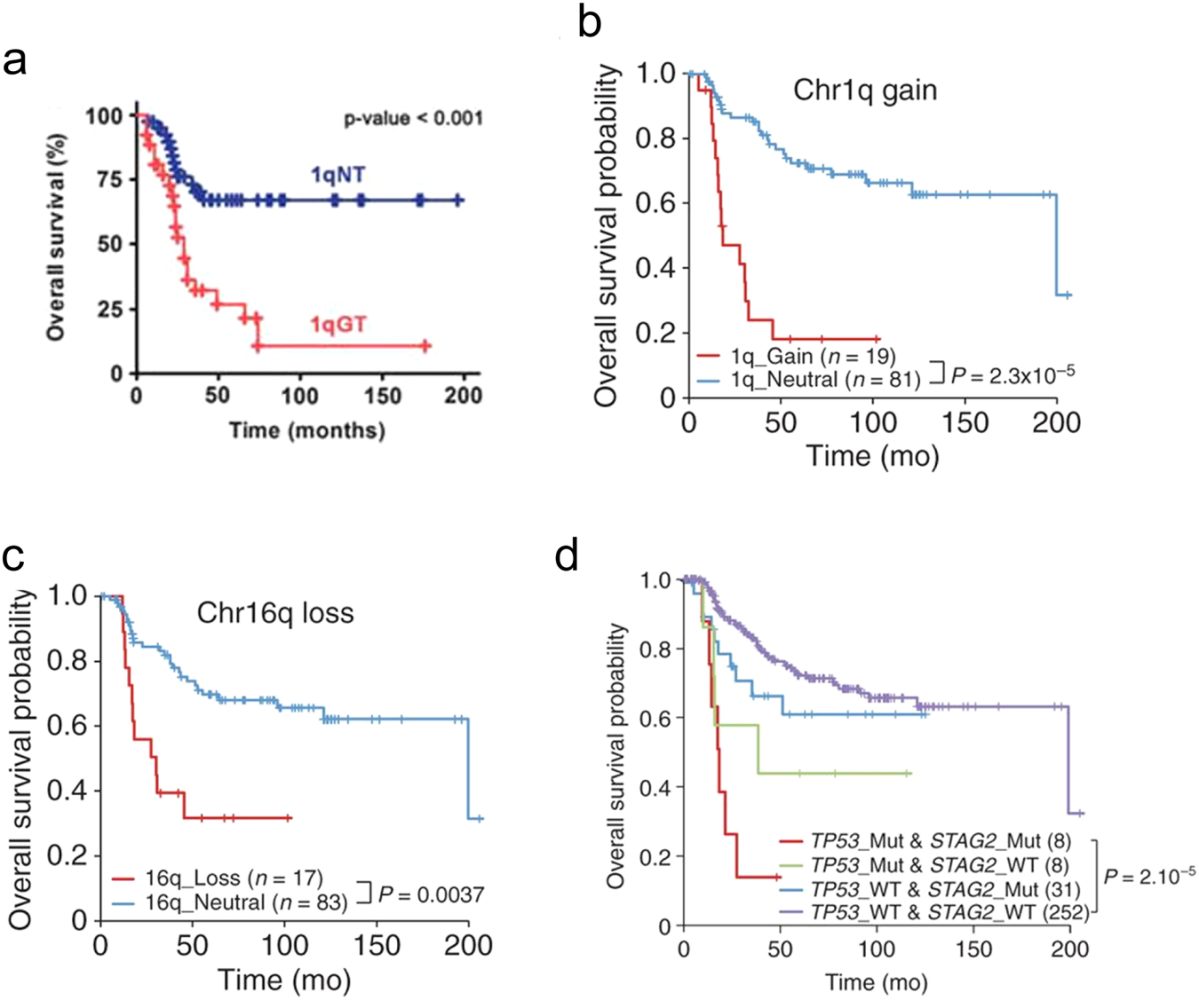
Poor prognostic molecular features of Ewing sarcoma tumors. Survival curves demonstrating the prognostic impact of 1q gain shown here are adapted with permission from Mackintosh C. et al., *Oncogene*, 2012^64^ (**a**) and Tirode F. et al., *Cancer Discovery*, 2014^65^ (**b**). Survival curves for 16q loss are shown here adapted with permission from Tirode F. et al., Cancer Discovery, 2014^65^ (**c**). Survival curves for patients stratified by STAG2 and TP53 status, adapted with permission from Tirode F. et al, *Cancer Discovery*, 2014^65^ (**d**).

Several studies investigating copy number variations (CNV) in EwS identified recurring abnormalities involving whole chromosomes or segments including gains in chr 8 (50% of cases), chr 2 (25% of cases), chr 1q (25% of cases), and chr 20 (10–20% of cases)^2,64^. The most common deletion involves chr 9p and *CDKN2A*^2^. Chromosome 1q gain is frequently associated with 16q loss as the result of an unbalanced t(1;16) rearrangement^64,65^. CNV studies within the EuroEwing 99/2008 trial have shown that 1q gain and, possibly, 16q loss define patients with adverse outcomes^64–66^. 1q gain was detected in 55% of relapsed specimens compared to 11.5% of non-relapsed and was associated with inferior OS (*P* < 0.001; Fig. 2a)^64^. A larger study verified this finding with 1q gain, demonstrating substantially inferior OS (*P* < 0.001; Fig. 2b)^65^. Similarly, loss of 16q was associated with inferior OS (Fig. 2c; *P* = 0.0037) and was co-associated with gain of 1q, although their combination did not show an additive effect on OS^65^. An attractive candidate on 1q is *CDT2*, a gene involved in cell cycle control whose gene dosage may increase proliferation rates in 1q gained EwS^64,67^.

Some studies have suggested prognostic significance of chr 8 gains, including whole chromosome, segment 8q, *MYC* (8q24) and/or *RAD21*.^68^ Chr 8 gain^69^ and *MYC* amplification^70^ have been suggested to be more common in relapsed tumors. A study of 52 EwS specimens showed gain in chr 8 or chr 12 in 83% of relapsed specimens compared with primary tumor (47%) or metastatic specimens (42%) from initial diagnosis^69^. In addition, chr 8 gain has been associated with a trend toward worse survival in two studies of 30 and 28 patients, demonstrating a non-significant decrease in 5-year EFS survival of 25–35% with chr 8 gain (*P* = 0.16).^71,72^ Studies from larger cohorts, however, have failed to demonstrate prognostic significance of chr 8 gains^65,66,73^.

In summary, CNVs are common in EwS and statistically significant differences in outcomes have been described, particularly for 1q gain and 16q loss. These prior studies are limited by patient numbers, and the lack of uniformity in the populations (localized vs. metastatic) and treatment approaches. In addition, variations in testing methodologies challenges the comparison of the results across studies. These results highlight the need to further investigate the prognostic significance of CNV at these loci prospectively by a uniform approach to truly define the landscape of these and other CNVs and their prognostic implications in EwS. Multiple studies are in progress with the most notable being a prospective validation of 1q/16q using 1q and 16q specific FISH probes that is currently being performed using the EuroEwing 2012 cohort.

### *TP53* mutations in EwS

*TP53* mutations have been identified in EwS tumor specimens collected at diagnosis and relapse and in ctDNA, using Sanger, whole genome, whole exome and panel sequencing methods^65,74–80^. Detecting functional alterations in p53 by immunohistochemistry has been historically difficult because pathogenic mutations can result either in loss of protein expression when *TP53* is deleted or truncated or high levels of nuclear protein accumulation in cancers with a missense mutation in *TP53*. Therefore, functional alterations of p53 are currently best detected by identifying pathogenic mutations by molecular laboratory methods.

Next-generation sequencing (NGS) studies of EwS cohorts demonstrated that pathogenic *TP53* mutations are detected in 5–10% of cases^65,74–77^. However, these studies included sample cohorts that were a mix of clinical phenotypes. Further studies are still needed to clearly delineate the rate of *TP53* mutations in patients with newly diagnosed localized disease, metastatic disease, and at the time of relapse. Associations between *TP53* mutations and outcomes in EwS have similarly been limited. Initial retrospective studies, including single-institution studies, suggested an association of *TP53* mutation with poor outcome^81–85^. However, a more contemporary study done in collaboration with the COG was unable to identify a significant association between pathogenic *TP53* mutations with outcome in patients with newly diagnosed localized EwS^86^. This study demonstrated non-significantly inferior outcomes for the 8 patients with *TP53* mutations (HR = 1.83 [95% CI: 0.65–5.19]) but was limited by cohort size (*n* = 96) and included sequencing of only *TP53* exons 5 through 8^86^.

### Loss of STAG2 in EwS tumors

STAG2 loss occurs in 15–20% of primary EwS^65,74,75^. Heterozygous somatic nonsense and frameshift mutations in the *STAG2* gene on the X chromosome were found to result in complete loss of protein expression, presumably due to inactivation on the other X chromosome in female patients.

The STAG2 protein is a component of the cohesin complex, which has a role in chromosomal organization and segregation. Recent studies now demonstrate, as previously noted, that loss of STAG2 expression in EwS alters the transcriptional program of *EWSR1::FLI1*, resulting in a more invasive cellular phenotype^87,88^.

Although *STAG2* mutations are readily detectable by many existing NGS panels, loss of STAG2 protein expression has been documented in tumors that do not have any detectable mutations in this gene. The evaluation of STAG2 expression may be better assessed through standard immunohistochemical (IHC) staining of tumor biopsy samples. Indeed, the binary nature of expression within EwS cells makes the interpretation of IHC staining relatively straightforward, even allowing for the identification of areas of subclonal loss of STAG2 expression in biopsy samples. Furthermore, the ubiquitous nuclear expression of STAG2 in endothelial cells, present throughout viable EwS tumors, provides an internal positive control on every stained slide.

A previous study demonstrated that *STAG2* mutation alone (*n* = 39) was associated with poor overall survival in a cohort of 299 patients with mixed clinical phenotypes (5-year OS ~50% vs. 70% for STAG2^mut^ vs. STAG2^WT^; *P* = 0.007)^65^. Loss of STAG2 expression by IHC was enriched in patients with metastatic EwS in a retrospective single-institutional cohort of 59 patients with newly diagnosed disease^75^. *STAG2* mutations also appeared to be acquired or selected for at the time of relapse^70,75^. Furthermore, by RNA-sequencing *STAG2* loss of function gene signature correlated with poor outcome^88^. A recent study of 108 patients with localized EwS previously treated on AEWS0031 demonstrated that STAG2 loss of expression occurred in 27% of patients, and 5-year EFS was 52% (95% CI 33–68%) and 75% (95% CI 63–84%) for patients with STAG2 loss vs. STAG2 expressed (*P* = 0.0018)^89^.

### Loss of STAG2 and *TP53* mutations

*STAG2* and *TP53* mutations co-occur more often than would be expected by chance^75^. In one study of 299 patients with EwS, the combination of *STAG2* loss and pathogenic *TP53* mutations was associated with a worse outcome than patients with either single variant alone and relative to wild-type *TP53* and *STAG2* (Fig. 2d; *P* = 2 × 10^−5^)^65^. In another study, one patient was found to acquire different sets of *STAG2* and *TP53* mutations at different recurrences suggesting a clonal advantage for cells with both variants^75^.

In summary, the clinical impact of *STAG2* and *TP53* mutations as potential prognostic biomarkers in EwS continues to rely on retrospective observational analysis. Prospective analyses of large clinically annotated cohorts in cooperative group studies will be required to fully test and validate their prognostic impact. Several ongoing efforts should provide the data needed to definitively determine the prognostic impact of *STAG2* and *TP53* in patients with localized EwS.

### Circulating tumor DNA

Detection of cancer-derived ctDNA from cell-free DNA isolated from bodily fluids, such as plasma, CSF, and urine has been utilized in multiple cancer types. In EwS, the most common detection strategy quantitates the pathognomonic *FET::ETS* fusions by either PCR or hybrid capture targeted NGS. PCR-based assays designed to detect fusion breakpoint sequences are highly sensitive^79,90–94^. However, the fusion breakpoints occur across broad intronic regions and are unique to each patient, requiring development of patient specific assays. Moreover, sequencing of tumor tissue is generally necessary for breakpoint identification. Targeted NGS panels of select *EWSR1* introns obviate the need for a priori tumor profiling but are not as sensitive as PCR-based assays. In a series of studies evaluating the ability to detect ctDNA in patients with newly diagnosed localized and metastatic patients with EwS, ctDNA detection rates for PCR- and NGS-based assays were 137/146 (94%) and 61/100 (61%) of samples, respectively^80,91,94–96^. In patients in whom ctDNA is detectable by both droplet digital PCR and targeted NGS assays, a strong correlation between the two methodologies has been demonstrated^79,96^. More recently, integrated methods including genetic and epigenetic detection of ctDNA has been applied to retrospective samples from patients with EwS, demonstrating highly sensitive and specific quantification of ctDNA.^78^ These methods show strong pre-clinical validation and require testing in larger clinical cohorts to determine clinical validity.

**Fig. 3:**
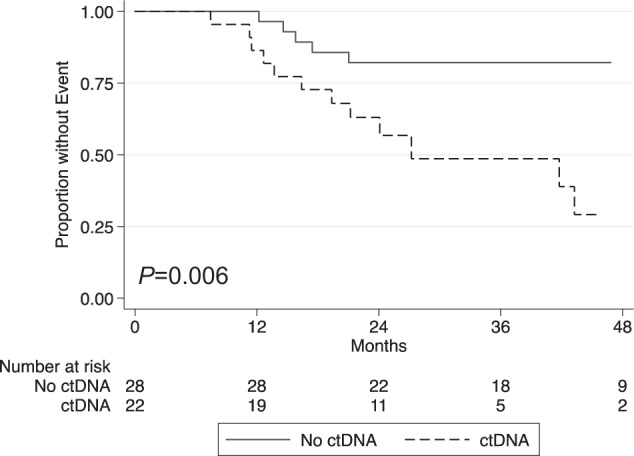
Prognostic significance of circulating tumor DNA (ctDNA) in Ewing sarcoma. Here we show that detectable ctDNA at diagnosis is associated with poor outcomes among patients with localized Ewing sarcoma. Adapted with permission from Shulman DS. et al., *BJC*, 2018^80^.

In one study of 50 patients with localized EwS, patients with detectable ctDNA by NGS (≥1.5%) had a 3-year EFS of 49% (95% CI: 24–69%) vs. 82% (95% CI: 49–93%) for patients without detectable ctDNA at diagnosis (Fig. 3)^80^. In a study of 102 patients with localized and metastatic EwS, ctDNA burden at diagnosis was divided into tertiles and higher ctDNA burden was associated with inferior outcomes^80,90^. However, these ctDNA tiers were not prognostic among the 67 patients with localized disease in that study. Serial assessment of ctDNA demonstrated that patients who remained ctDNA positive at start of chemotherapy cycle 2 and 3 were more likely to have relapse events^90^.

Given the well-established ctDNA technology and strong preliminary data, prospective ctDNA-based biomarker studies should be incorporated into clinical trials for EwS as an integrated biomarker to confirm the prognostic relevance of specific ctDNA levels at diagnosis as well as the predictive value of ctDNA during treatment. Importantly, the translation of these research-based assays into a clinical laboratory environment will be needed if ctDNA analyses are to be utilized for risk-stratification. Given the long lead time required for clinical implementation of NGS and/or patient-specific MRD assay platforms, consideration should be given to starting this process now.

### Tumor mutational burden

Ewing sarcoma (EwS) is characterized by a remarkably quiet genome, with a low TMB relative to most other malignancies^97^. While recurrent mutations have been described in *STAG2* and *TP53*^65,74,75^ (see earlier), the overall mutational burden across the genome in these tumors is generally less than 1 mutation/Mb and nearly always <10 mutations/Mb^62,97,98^.

In this context, two studies have evaluated the potential prognostic impact of TMB in EwS. The first study utilized whole genome sequencing (WGS) to classify patients into tertiles defined by SNVs and indels, with a statistically significant association with overall survival^65^. However, the effect size was relatively modest with 5-year overall survival estimates clustered within 20% points across all tertiles. Another group utilized WGS data and an analytic algorithm that provided an estimate of mutations that led to protein alterations^99^. When analyzed in this way, patients with higher mutation burden had statistically significantly inferior overall survival, with a univariate hazard ratio of 2.6. Higher mutation burden was associated with older age and metastatic stage, though mutation burden remained prognostic on multivariate analysis.

Overall, the main limitations of utilizing TMB for clinical risk stratification are the paucity of data supporting this approach, the lack of substantial dynamic range in the marker, and the relatively modest effect size associated with this marker.

### Germline DNA damage defects

Historically, unlike some other subtypes of pediatric sarcomas, EwS has not been associated with classic cancer predisposition syndromes^2^. Exceedingly rare reports (*n* = 3) exist of siblings developing EwS^100,101^. In one study, first-degree relatives of patients with EwS have an increased risk of developing cancers such as brain and female genital cancers, and second-degree relatives demonstrated an increased risk of breast cancer among others^102^. Genome-wide association studies have identified genetic haplotypes associated with an increased risk of EwS^103,104^, which possibly contribute to disease onset through generating higher affinity binding sites for EWSR1::FLI1 as shown at *EGR2* cis-regulatory elements^105,106^.

In the past ~5 years, an emerging subset of patients with EwS and pathogenic germline variants in genes involved in DNA damage repair have been noted, due in large part to multiple large-scale sequencing efforts in the pediatric oncology population and the increase in patient tumors being sent for sequencing upon relapse^107–114^. Examples of genes impacted in this patient cohort include but are not limited to *FANCC*, *FANCM*, *BRCA1*, *BRCA2*, *RAD51*, *BARD1*, *SLX4*, and *PALB2*. While any individual gene variant is rare, as a group, these pathogenic germline variants are found in ~10–13% of patients with EwS.

Clinically, this subset of patients is of high interest given the possibility that these patients may respond differently to therapy as compared to patients without an additional deficit in DNA damage repair. Intriguing future questions to address include: (1) Are overall patient outcomes different? (2) Would this cohort be more likely to respond to certain DNA damaging agents/combinations in the setting of relapse? (3) Does this cohort experience more toxicities from treatment? (4) If outcomes are better in this cohort, should a study of therapy reduction be considered?

When considering the future of risk-stratifying patients with EwS, patients with pathogenic germline variants in DNA damage repair genes are a logical group of patients to study prospectively on future clinical trials.

## Future directions

### Justification for tissue collection

These studies together highlight molecular features that may stratify patients into higher and lower risk therapeutic treatment cohorts. The limitations of most published studies include sample size and challenges in integrating data across studies due to the non-homogeneous mixture of patient treatment protocols and variations in the molecular approach to analysis. Centralized tissue collection and analysis would mitigate many of these issues. Indeed, centralized translocation testing is justified based on the nuanced approach to diagnosis described above and the need to exclude patients with alternative fusions such as *CIC::DUX4*, or cases with *EWSR1::non-ETS* fusions that would appear as EwS if only a break-apart FISH is used. Other promising biomarkers such as *STAG2* and *TP53* require state of the art IHC and genomic analyses to identify all the various pathogenic mutations. Further, the detection of copy number changes and the quantitation of ctDNA can each be performed with multiple techniques. Therefore, uniformity in the analysis is required before statistically valid and biologically meaningful conclusions can be drawn about any of these biomarkers. A centralized approach would provide high-quality, outcome-linked, standardized tissue collection that will facilitate further pre-clinical analysis of these and other molecular features as technologies and science evolve. The hope is that this will drive discovery of more effective and less toxic precision therapy.

### Inclusion of centers large and small

Marked disparities in access to, and quality of care contribute to differences in prognosis and outcome for patients who are afflicted with serious illnesses, including cancer^115^. If not recognized and mitigated, these disparities can be especially profound for patients who suffer from rare diseases. Given their relative rarity, sarcomas, including EwS, present diagnostic challenges and centralized pathology review has been shown to greatly increase diagnostic accuracy in several cooperative sarcoma studies^116–118^. In the US, the NCCN guidelines now recommend diagnosis at an institute with access to NGS testing. In addition, patients with EwS receive complex multi-disciplinary care that most often requires travel to an urban center, a factor that amplifies inequities^116^. Moreover, given the unique challenges faced by AYA patients, the increased prevalence of EwS in this under-represented patient population compounds the potential for disparities in both access and outcomes^119^.

As our knowledge of fundamental biologic processes and targeted therapeutics grows, the critical role of advanced molecular tools for the diagnosis and treatment stratification of sarcomas, including EwS, becomes increasingly important^2,120^. As detailed in this review, there are many new and innovative biomarkers that may, with further study, prove useful for prognostication in newly diagnosed and relapsed patients. If so, this will enable the development of new clinical trials and treatment strategies that could finally improve survival for patients with high-risk disease and lessen unnecessary treatment-related toxicity for low-risk patients. To ensure that all patients with EwS benefit from these advances, it will be essential that access to these innovative diagnostic and prognostic tools be available to all patients. The centralized nature of pediatric cancer care in most high-income countries, alleviates some disparities, though the need to travel to large urban centers remains a major inequity^121^. Therefore, it is an ethical imperative that the future testing, validation, and implementation of prognostic and predictive biomarkers be achieved through an equity lens. As such, we propose that the need for equal access to centralized pathologic review and tumor testing should be considered as essential elements of any new assay or approach.

### Clinical trial integration

A refined risk-stratification approach to EwS inclusive of biologically relevant molecular features would have substantial implications for clinical trial design, especially in the localized patient population. There exists sufficient prognostic information and readily available assays to justify prospective evaluation of multiple biomarkers, as pre-specified trial aims, in all patients to define biologically relevant subtypes.

**Fig. 4:**
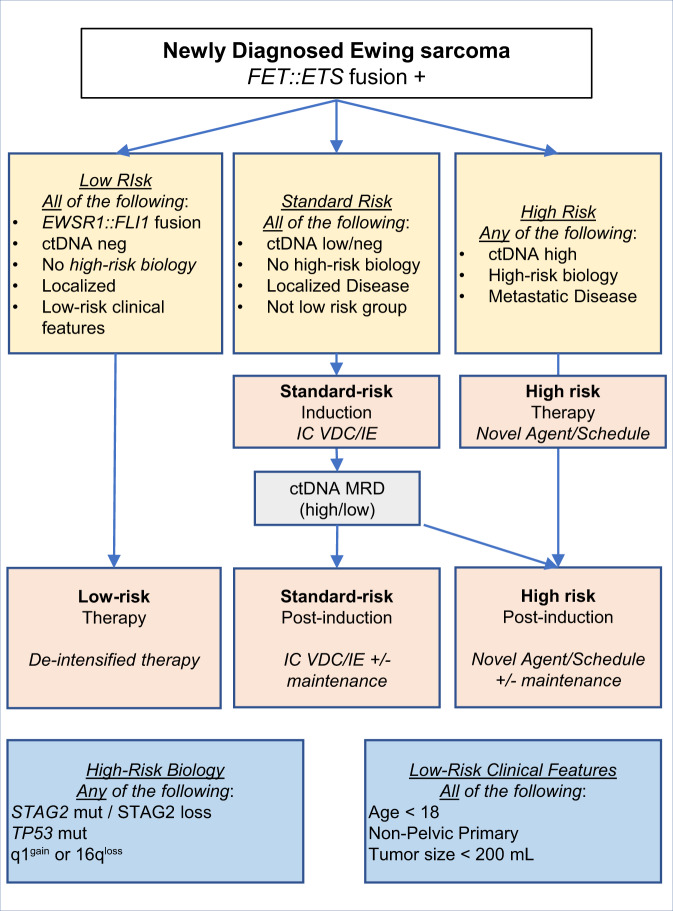
Hypothetical risk-stratified treatment schema. We provide a hypothetical risk-stratification schema that one could envision as the biomarkers described in this manuscript are validated. This schema incorporates clinical and molecular biomarkers.

As the above molecular biomarkers undergo further study, one could consider how they might be incorporated into a future risk-adapted frontline clinical trial should these biomarkers be successfully validated. Here, we propose a hypothetical schema based on available data that incorporates both clinical and molecular features. The schema utilizes three risk groups: a low-risk, standard-risk and high-risk group. The low-risk group would include patients with an estimated 5-year EFS of ≥90%. Reasonable approaches to de-escalation would include reduction of alkylators, doxorubicin or etoposide to reduce fertility, cardiac, and second malignant neoplasm-related late effects. The standard risk-group would include patients with an estimated 5-year EFS of 50–90% who should continue to receive current standard risk chemotherapy (i.e., interval compressed VDC/IE) with or without additional trial interventions of minimal risk, such as maintenance therapy. The high-risk group would include patients with a <50% 5-year EFS and receive the standard chemotherapy backbone with the addition of a targeted agent and/or novel approaches to extending or intensifying chemotherapy (Fig. 4). Beyond prognostication, the promise of serial biomarker assessments and response-based interventions including changes in ctDNA, and tumor volume, paired with examination of tumor tissue viable populations after induction chemotherapy should be incorporated into trials^78,80,94,122^. Given that most patients with EwS go into a radiographic remission prior to disease relapse, these efforts must prioritize understanding molecular minimal residual disease. Collection of specimens from patients with disease relapse must also be prioritized to improve our understanding of mechanisms of resistance. Translational teams should determine the best, non-consumptive, correlative studies to evaluate mechanisms of resistance and relapse to advance our understanding of tumor evolution and acquired resistance with epigenetic changes from *STAG2* in particular^87,88,90,123^.

## Conclusions and perspective

In summary, we have assembled an international working group of disease experts to review available molecular biomarkers with the potential to be used in combination with clinical factors to identify disease subgroups. Many of the molecular biomarkers covered in this review have strong preliminary evidence as prognostic biomarkers in EwS. Translocation was the only biomarker with strong enough evidence to warrant use as an integral biomarker for enrollment to future clinical trials for the purposes of identifying patients with EwS, separate from *EWSR1* round cell sarcoma with non-ETS fusion partners, *CIC::DUX4* sarcomas and *BCOR::CCNB3* sarcomas. Translocation type and chromoplexy remains an area of active investigation and requires further study before being used for risk-stratification. Co-occurrence of 1q^gain^ and 16q^loss^ have strong preliminary evidence and are currently being validated on the EuroEwing 2012 trial prospectively. Similarly, *STAG2* is the most prognostically-valuable single gene candidate and is being evaluated in EuroEwing 2012 trial and in a large cohort of previously treated patients from AEWS1031 and AEWS0031, alone and when co-mutated with *TP53*. ctDNA is the most promising peripheral blood biomarker, potentially representing a measure of micrometastatic disease, with preliminary evidence from two large cohorts from the United States and Europe. Prospective validation of diagnostic ctDNA burden is underway. We note that while our review was focused, many other promising prognostic biomarkers beyond this review have been proposed, including but not limited to SOX2, mir-34a, Ki67, neurexin-1, RRM2, PRC1, IGF1/IGFBP3, and may also warrant further evaluation^124–131^.

These biomarkers, primarily studied over the prior decade, represent promising markers for testing of risk-adapted treatment approaches but require definitive validation prior to use for assigning therapeutic strategies. These studies will provide the first step in the realization of risk-adapted treatment strategies. Therefore, our review highlights the need for comprehensive evaluation of these biomarkers in large, annotated cohorts with pre-planned analysis such that the prognostic impact of each marker can be definitively determined in the context of relevant clinical features. Such efforts are ongoing and will inform future attempts to implement risk-adapted therapy. The ultimate success of these strategies will be greatly enhanced through collaborative science and international harmonization of approaches to biomarker implementation.

## Supplementary information

Supplemental Table 1
