# Supplementary material for: An international working group consensus report for the prioritization of molecular biomarkers for Ewing sarcoma
Source: NPJ Precis Oncol. 2022 Sep 17;6:65. doi: 10.1038/s41698-022-00307-2 (PMC9482616; doi:10.1038/s41698-022-00307-2)
Supplement: Supplementary file 1 — Supplemental Table 1 [file 41698_2022_307_MOESM1_ESM.docx]

**Supplemental Table 1**: Relevant clinical trials, evaluated therapy and outcomes. Adapted from Zollner et al., *J Clin Med*, 2021^1^

| **Trial Name** | **Chemotherapy Backbone**  **Evaluated** | **Key Findings** |
| --- | --- | --- |
| AEWS0031 | IC-VDC/IE | Six-year EFS improved to 73% from 65% with interval  compressed chemotherapy for  **localized** EwS |
| EURO-E.W.I.N.G.  99 and Ewing 2008 | VAI vs. VAI/HD-BuMel | Eight-year EFS improved to 60.7% from 47.1% for **localized** high-risk EwS |
| AEWS1031 | VDC/IE vs. VDC/IE/VTC | No benefit to the addition of VCT cycles for **localized** EwS |
| AEWS1221 | VDC/IE vs. VDC/IE/ganitumab | No improvement in outcomes for **metastatic** EwS with the addition of ganitumab |
| EURO Ewing 2012 | VIDE induction +  VAI/VAC (or VIA/HD-BuMel) vs. VDC/IE induction +  IE/VC (or VAI/HD-BuMel) | VDC/IE induction was found on preliminary analysis to have superior PFS and OS compared to VIDE induction |

1 Zollner, S. K. *et al.* Ewing Sarcoma-Diagnosis, Treatment, Clinical Challenges and Future Perspectives. *J Clin Med* **10**, doi:10.3390/jcm10081685 (2021).
